# Assessment of Data Usage of Cancer e-Interventions (ADUCI) Framework for Health App Use of Cancer Patients and Their Caregivers: Framework Development Study

**DOI:** 10.2196/18230

**Published:** 2020-09-15

**Authors:** Natalie Heynsbergh, Seung Chul (Eric) O, Patricia M Livingston

**Affiliations:** 1 Faculty of Health Deakin University Geelong Australia

**Keywords:** multimedia, user engagement, cancer, smartphone, framework, usage data, eHealth technology, e-intervention, data analysis, efficiency, e-research, apps

## Abstract

**Background:**

Multimedia interventions can provide a cost-effective solution to public health needs; however, user engagement is low. Multimedia use within specific populations such as those affected by cancer differs from that of the general population. To our knowledge, there are no frameworks on how to accurately assess usage within this population to ensure that interventions are appropriate for the end users. Therefore, a framework was developed to improve the accuracy of determining data usage. Formative work included creating a data usage framework during target audience testing for smartphone app development and analysis in a pilot study.

**Objective:**

The purpose of this study was to develop a framework for assessing smartphone app usage among people living with cancer and their caregivers.

**Methods:**

The frequency and duration of use were compared based on manual data extraction from two previous studies and the newly developed Assessment of Data Usage of Cancer e-Interventions (ADUCI) Framework.

**Results:**

Manual extraction demonstrated that 279 logins occurred compared with 241 when the ADUCI Framework was applied. The frequency of use in each section of the app also decreased when the ADUCI Framework was used. The total duration of use was 91,256 seconds (25.3 hours) compared with 53,074 seconds (14.7 hours) when using the ADUCI Framework. The ADUCI Framework identified 38 logins with no navigation, and there were 15 discrepancies in the data where time on a specific page of the app exceeded the login time. Practice recommendations to improve user engagement and capturing usage data include tracking data use in external websites, having a login function on apps, creating a five-star page rating functionality, using the ADUCI Framework to thoroughly clean usage data, and validating the Framework between expected and observed use.

**Conclusions:**

Applying the ADUCI Framework may eliminate errors and allow for more accurate analysis of usage data in e-research projects. The Framework can also improve the process of capturing usage data by providing a guide for usage data analysis to facilitate evidence-based assessment of user engagement with apps.

## Introduction

### Background

Within the health care context, electronic health (eHealth) technologies such as smartphone apps can assist people in managing their health by providing information, support, communication, and resources to track the progression of well-being or illness [1]. In 2017, over 318,000 health apps were available to download [2]. Evidence-based apps and formal evaluations are growing in chronic disease areas such as for diabetes and mental illness, including anxiety and depression [2]. However, there is little evidence on app usage within the adult cancer population [3-5].

Worldwide, there is a need for supportive eHealth technology in the cancer field as the provision of cancer care has shifted toward an outpatient setting [6]. The adoption of human-computer interaction and user-centered design principles can guide intervention development to inform user needs [7]. User-centered design approaches have been adopted to develop two apps: one for people living with cancer [5] and one for caregivers [4]. User-centered design enables developers to identify the unique needs and behaviors of population groups to ensure that technology accurately reflects users’ requirements [7]. Previous technology-based strategies have been used in the cancer field to promote emotional well-being such as audio-visual techniques, and have used a similar approach of seeking user engagement to guide the development of new interventions [8].

Many adults living with cancer and their informal caregivers are managing cancer in the community; thus, the need for cost-effective supportive interventions is vital to inform patient and caregiver care needs. Smartphone app interventions have the potential to be more cost-effective than face-to-face interventions; however, this can depend on adherence and use [9]. Positive engagement of users directly impacts users’ motivation and intention to use multimedia platforms and apps [10]. There is no single definition or concept of user engagement; rather, its complexity involves the investment of a person into using a program and encompasses satisfaction, ability to engage, and sustained engagement [11]. Within the general population, user engagement with eHealth interventions is low; over an 18-month surveillance period, engagement with self-guided apps ranged between 13% and 26% [12]. In the adult cancer setting, usage of apps is relatively unknown with few evaluations having been completed [4,5]. App usage among this population may also differ from that of the general population as patients and caregivers are usually highly burdened, distressed, and lacking in time [13,14]. Therefore, it is unknown what constitutes “active engagement” in this group. Therefore, frameworks to measure use and engagement in the adult cancer setting are required to facilitate the accurate evaluation of interventions for informing the feasibility and cost-effectiveness of evidence-based apps. To our knowledge, no frameworks addressing this topic exist. We performed a scoping review of the literature and consulted leading eHealth specialists across the state of Victoria in Australia, and no similar frameworks or guidelines were identified.

To fill this gap, the Assessment of Data Usage of Cancer e-Intervention (ADUCI) Framework was developed and validated using a three-step approach. Step one involved creating a framework for user testing during development [15]. In step two, the framework for app use and engagement was developed by analyzing findings of a pilot study to determine the feasibility of app interventions [4]. We here report the results of step three, in which the framework from the pilot study [4] was corroborated by applying it to another study in a randomized controlled trial [5].

### Study 1: User Testing

An app usage framework was developed during the planning, design, and evaluation phases of an app for cancer caregivers. This comprised two phases, user acceptance testing and user experience testing, during app development [15]. Participants were provided with scenarios and were required to find the corresponding information within the app. Each scenario was timed and a cutoff of 20 seconds was applied to guide the time necessary to complete tasks. The timeframe of 20 seconds was used as a guide from the general population and was amended to inform use of an app among older adults [16]. Tasks that took participants over 20 seconds to complete or were incomplete resulted in corresponding content and design changes to the app.

### Study 2: Pilot Study Usage Data Analysis

Adult caregivers of people with colorectal cancer receiving chemotherapy in the outpatient setting were approached and invited to participate in the feasibility pilot study. Caregivers who participated in the study were provided with access to the “Carer Guide” smartphone app for 30 days. Carer Guide included access to information and resources to help manage the needs of people with colorectal cancer as well as the caregivers’ own needs. Caregivers were required to log in to access any information within the app. Data tracking was recorded using Google Analytics, and included the frequency of login, length of login, and number of pages visited. The ADUCI Framework was validated in this study by applying data cleaning methods to analyze and report usage data. On average, caregivers used the app for 22 minutes each time they logged in [4].

### Ethical Concerns

Both studies were approved by the Human Research Ethics Committees at the relevant health care organizations and at Deakin University. Participants were informed in writing that usage data would be monitored to determine which pages were visited.

### Objectives

The aim of this study was to corroborate the ADUCI Framework by applying it to data usage from a randomized controlled trial (study 3) involving a smartphone app for people diagnosed with cancer [5].

## Methods

### Study 3: Applying the ADUCI Framework to a Randomized Controlled Trial

Patients receiving chemotherapy in an outpatient setting in Melbourne, Australia were approached and invited to participate in the study. Following recruitment, participants were randomly allocated to either the intervention or control group. Participants in the intervention group had access to a smartphone app (“ACE” app), which provided static information and support resources to participants [5]. ACE app resources included the ability to view and change hospital appointments and record notes in a notebook. Over a 4-month period, participants could access ACE when needed, and received monthly reminders to complete distress thermometer scores. Participants could access information and support freely; however, they had to log in to the app to change appointments and complete distress thermometer scores. Participant usage data were tracked internally through the ACE app. Tracking information included login frequency, duration of login, and number of pages visited.

### Assessment of Data Usage With the ADUCI Framework

The ADUCI Framework comprises two components: assessment of duration of use and frequency of use. Duration of use was measured as the length of use in seconds for each app login, as well as the amount of time spent in each section of the app. Frequency of use included the number of logins overall and the number of times each section of the app was visited.

#### Framework for Duration of Use

Based on the findings of the pilot study [4], a 22-minute cutoff was applied for each login of the ACE app after page navigation had ceased. The purpose of this cutoff was to standardize the usage data that were manually extracted and to remove or reduce situations where people may have the app running in the background on their phone without actively using the app. Similarly, app usage less than 1 second was not included in the analysis, as 1-second use often had no navigation and may have been a user error in clicking on the wrong app icon.

#### Framework for Frequency of Use

The frequency of page visits was cleaned and analyzed in the following format. When navigation moved from a content page to the main menu and back to the same content page (eg, *Cancer Information, Main Menu, Cancer Information*), the frequency of use of the *Cancer Information* page was interpreted as 1 (Scenario 1). When navigation moved between several pages but page visits were repeated, the page visits were tallied (Scenario 2). For example, when navigation followed the order *Cancer Information, Main Menu, Wellbeing, Main Menu, Cancer Information*, this was interpreted as 2 views for *Cancer Information* and 1 view for *Wellbeing.*

These rules were applied since in Scenario 1 there was no information as to whether this navigational pattern was intentional or accidental, whereas in Scenario 2, as another page had been visited in between the two times that the *Cancer Information* page was visited, subsequent use of this section of the app was most likely intentional.

In the event that users logged into the app for longer than 1 second but had no navigation beyond the main menu, this event was removed from the analysis as no use of app content occurred.

**Figure 1:**
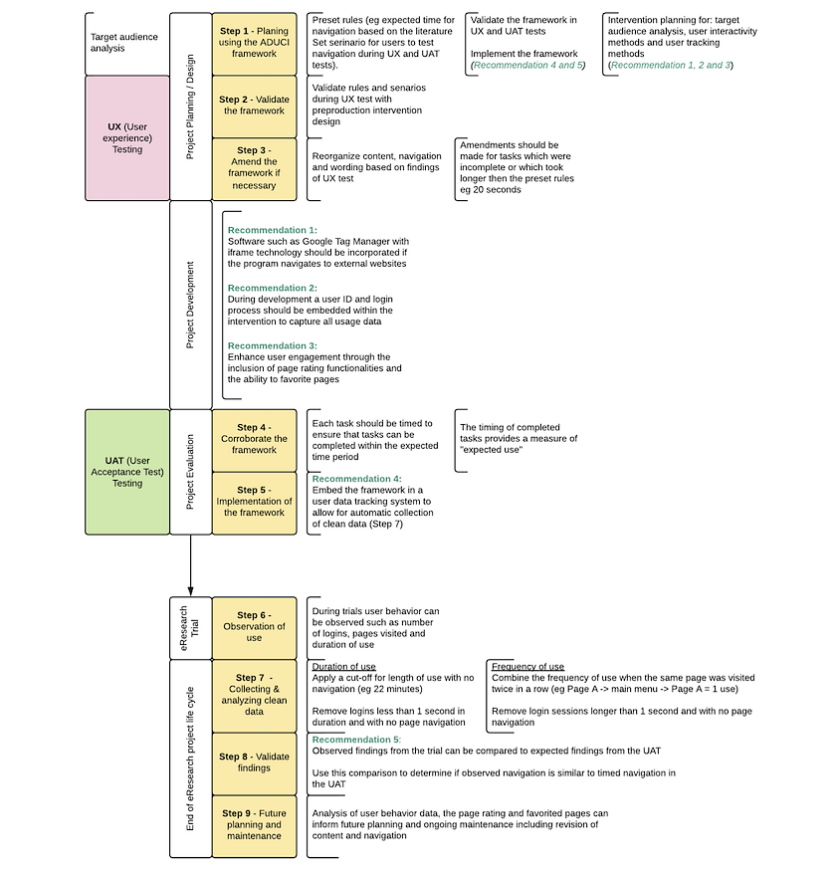
The Assessment of Data Usage of Cancer eInterventions (ADUCI) Framework and future recommendations for eInterventions.

The ADUCI Framework includes nine steps to guide intervention planning, development, user testing, data cleaning, and analysis. Future recommendations outlined in the Results section below have also been incorporated into the process. See Figure 1 for an overview of the framework.

### Data Analysis

Descriptive statistics were used to summarize the duration and frequency of use of each login and each section of the app used. Data were analyzed by comparing results from the manual extraction and when the ADUCI Framework was applied.

## Results

### Frequency of Use

A total of 279 logins were recorded in the manual extraction of data compared to 241 when using the ADUCI Framework. The mean difference in number of logins was 6 (range 0-13) when comparing manually extracted data with the ADUCI findings (Table 1). The most frequently used sections of the ACE app had the highest number of discrepancies between manual data extraction and when the Framework was applied. Frequency of use was similar between the manual extraction and the ADUCI Framework; however, the Framework showed a reduction in frequency of use in each section of the ACE app (Table 1).

**Table 1:** Frequency of use of the ACE app and each section within the app over a 4-month period.

App feature accessed	Manual extraction (n)	ADUCI^a^ Framework (n)	Difference
Cancer information	70	57	13
Navigation	40	33	7
Allied health	21	13	8
CCV^b^ support service	29	28	1
Clinical trials	28	23	5
Appointments	165	159	6
Need help	7	3	4
About us	0	0	0
Notepad	42	35	7

^a^ADUCI: Assessment of Data Usage of Cancer Electronic Interventions.

^b^CCV: Cancer Council Victoria.

### Length of Use

The manual extraction highlighted that, on average, the ACE app was used for 328 seconds at each login, and for a total of 91,256 seconds over the 4-month intervention period (Table 2). When the ADUCI Framework was applied, the mean length of use was 224 seconds and total use was 53,074 seconds. Across the different sections of the app, the total length of use ranged from 0 seconds to 213,930 seconds (3566 minutes or 59.4 hours) in the manually extracted data compared to 0 to 52,074 seconds (230 minutes or 3.8 hours) when the ADUCI Framework was applied.

**Table 2:** Length of use of the ACE app in seconds.

App feature accessed	Manual extraction			ADUCI^a^ Framework			Difference	
	Mean	Total	Mean		Total	Mean		Total
All uses	328	91,256	224		53,074	104		38,182
Cancer information	3450	213,930	266		13,813	3184		200,117
Navigation	2376	80,784	74		2897	2302		77,887
Allied health	56	1190	56		890	0		300
CCV^b^ support service	22	559	125		2996	–103		–2437
Clinical trials	1358	38,018	25		628	1333		32,390
Appointments	1142	161,010	71		10,434	1071		150,576
Need help	25	178	30		150	­–5		28
About us	0	0	0		0	0		0
Notepad	1243	45,976	81		2686	1162		43,290

^a^ADUCI: Assessment of Data Usage of Cancer Electronic Interventions.

^b^CCV: Cancer Council Victoria.

Application of the ADUCI Framework resulted in a reduction of time spent in each section of the app, with the exception of the Cancer Council Victoria (CCV) support service. During data cleaning, additional time was recorded in the CCV support service section (Table 2).

In the manual extraction, there were several errors in the data recorded. Errors showed that the duration of use of a specific section of the app exceeded the duration of login. For example, the *Cancer Information* section was recorded as having been used for 16,072 seconds, whereas the total login duration of the app for that session was recorded as 4 seconds. Similar discrepancies occurred 15 times in the manually extracted data. These discrepancies were eliminated when the ADUCI Framework was applied.

### Future Work

This analysis informed the development of a framework for analyzing user usage data during the project planning/design and evaluation stages. In addition to the proposed ADUCI Framework, there are four recommendations for future eHealth interventions, which can provide a more effective approach to handling usage data as well as implementing user engagement methods to reduce the time needed for manual data cleaning. The ADUCI Framework offers a more accurate approach of analyzing usage data; however, there were gaps present in the data collected, and additional features and functionality would ensure that complete data are available for analysis. These features and functionalities are listed below as future recommendations.

#### Recommendation 1: Track All Usage Data if Possible

Due to the sensitive nature of health information and its potentially huge storage and maintenance requirements, most eHealth mobile apps cannot contain all of the information required by end users. Quite often, apps use linking methods to specific information outside the app. Researchers and developers should consider all possible issues with tracking usage data during the planning and design stage of project development to apply a suitable solution to avoid missing usage data. In the development stage, internal and external testing should be conducted at various times with small groups of end users to ensure that the system collects all intended usage data.

An example of this situation within the ACE app is the feature that linked patients to external CCV webpages. In both the pilot study [4] (study 2) and the ACE app trial [5] (study 3), the tracking system was unable to continue monitoring data usage once users navigated out of the app ecosystems to external websites. This issue can be solved by using a technical solution such as Google Tag Manager with iFrame technology to link and track usage outside the app.

Using a solution such as Google Tag Manager with iFrame technology will address two gaps in user engagement analysis. First, it will provide full coverage of end user data usage and behavior, allowing for more accurate assessment of usage data. Second, it will provide developers with a complete understanding of what the end user needs and be able to provide tailored content in current or future projects.

 

#### Recommendation 2: Link User ID to Tracking Record

Similar to the ACE app, across other health conditions, it is common to find that user IDs are not incorporated into the development of apps [17]. A login should be present to enable tracking of all of a user’s usage. This may be achieved via the functionality of having people log in once and enabling a “remember me” function on the app. This function should be considered in the planning stage. In addition, if the app does not have prior logins recorded to use, it should request that the user log in before continuing to access the app.

However, this functionality needs thorough internal and external testing during the app development and evaluation stages to ensure that it is working properly, and that all relevant data are being captured.

Similarly, the decision of whether or not to implement a user login process within apps is an important consideration. A limitation of the ACE app was the loss of user data throughout the trial, as users were only required to log in to change appointments and record distress thermometer readings [5]. Minimizing security requirements such as logins can increase the accessibility of apps as users are not required to remember login details; however, without these security measures, it is not possible to definitively track user data. Incorporating user IDs should be balanced between the characteristics of the end users, the purpose of the app, and the need to provide accurate usage data.

#### Recommendation 3: Enhance User Engagement

E-interventions and e-research projects are often developed for specific end users. Therefore, it is essential to undertake target audience analysis to understand the audiences’ needs, and to design user engagement tools based on concepts of human-computer interaction.

User-centered design is closely aligned with human-computer interaction to ensure that programs are developed to meet the needs and capabilities of users [7]. Both apps in this study were iteratively tested using a user-centered design approach to ensure that the content, functionality, and usability were suitable. Findings between studies were similar where the majority of participants were female, in their 50s, held a tertiary-level qualification, and used a smartphone. Based on these demographic characteristics, we suggest designing a user interface with similar characteristics to popular social media platforms, and providing functions that give users the ability to fully control and personalize program content to enrich their experience [18-20].

User engagement with interventions and app popularity may be enhanced using the following tools: (1) star rating functionality, as a useful measurement of content and interactive media presence [21]; (2) interactive media favorite functionality, for building an individual end user’s custom interactive content and media library [22]; (3) counting the star rating when observing other users’ activities; and (4) providing a familiar interface such as consistent design with current social media and networking web content so that engagement strategies (eg, star rating) are familiar [23,24].

These engagement tools will provide strong evidence-based guidelines for program content development in the future by highlighting which content is most highly rated, most favorited, and most respected. Such tools may also provide evidence as to which existing content needs attention from the program content developers. Developers should evaluate the engagement strategies with users at the end of the project life cycle to build better engagement tools for future studies.

#### Recommendation 4: Implementation of the ADUCI Framework in the System

Usage data findings presented in the data collection and data analysis phase of ADUCI Framework development emphasize the importance of having frameworks to properly assess and manage usage data throughout the life cycle of development and testing of e-interventions. If implemented in the evaluation stage of an e-research project development life cycle, usage data can be more effectively analyzed. For example, it required approximately 40 hours to manually extract data from the ACE app study to determine a more accurate and useful dataset. By implementing the practice recommendations and ADUCI Framework, the final analysis of returned usage data will be performed much more efficiently, with both time and cost advantages.

#### Recommendation 5: Validate Findings and Provide Opportunities to Map User Behavior

This paper provides an overview of the ADUCI Framework by comparing findings between manually extracted data and data obtained using the Framework. However, there was limited ability to assess the accuracy of user behavior and pathways data from this study. In future studies, developers and designers could conduct more specific target audience analysis to understand specific behaviors for building a tailored framework in the planning stage. The Framework could be validated by comparing expected use in different stages of the project. In the project planning stage, user acceptance testing and user experience testing could include scenarios and measurement methods for expected use. In the design, development, and evaluation stages, use could be observed and measured during user testing. After the launch of the project, usage data could be obtained through clinical trials. This comparison of expected versus observed use may also provide more opportunity to assess user mapping and determine pathways of user behavior.

#### Recommendation 6: Ethics and Transparency With Users

To ensure that vulnerable populations such as people with a cancer diagnosis are protected, users should be informed of the intent to collect usage data as proposed in this Framework. Clear identification of the type of data intended to be collected and the purpose for data collection is vital in promoting trust between users and app developers. Particularly within clinical trials, this can be achieved during the information and consent process to ensure that users are providing informed consent. In both of the studies used to develop this Framework, ethics approval was obtained and participants were informed of usage data tracking prior to providing consent. Usage data were collected to inform the evaluation of and engagement with the app interventions in line with a user-centered design approach, allowing for future iterations to build upon findings. To protect users’ privacy, only coded data were extracted that were linked to user IDs. No personal or identifying information was obtained or used.

### Next Steps for the ADUCI Framework

The ADUCI Framework facilitates the extraction of accurate usage data and informed approaches to reduce the burden of lengthy manual data extraction. However, the Framework has limitations because it is in the early stages of development. The proposed recommendations create the next opportunity to test and refine the Framework, and to assess user engagement based on tailored comprehensive target audience analysis. Recommendations 1 and 2 will prevent usage data leakage while allowing developers to understand target audience behavior, thereby providing more accurate usage data for analysis. Recommendation 3 will give target audiences enriched personalization to engage with the program. The collectable data from personalization methods, including star rating, favorite, and counting star rate functionalities, will supply evidence-based future planning sources. Recommendations 4 and 5 will allow for the development of a more robust framework for projects and will provide the opportunity to validate the Framework.

## Discussion

### Principal Findings

Applying the ADUCI Framework can reduce errors and allow for a more accurate analysis of usage data. The ADUCI Framework describes how to incorporate and analyze usage data at each stage of the research life cycle, including development during user testing, pilot testing, and applying findings to a randomized controlled trial. This provides future research with a framework for measuring and testing functionality, usability, and user engagement.

The importance of precisely reporting usage data is to provide an accurate representation of user engagement with e-interventions. Consideration of user engagement begins during the planning and design stages, and continues through the development and evaluation stages of an e-research project’s life cycle. Enhancing user engagement includes developing programs that are quick, easy, and intuitive to use [25]. This process was achieved and addressed during user testing, and included having a cut-off time of 20 seconds to complete actions. This cut-off time allowed for any changes to be made to ensure that the content was easily navigated and functionality was usable by the targeted audience.

User engagement continues to be monitored and assessed throughout the life of interventions. Satisfaction with interventions can have a positive impact on user engagement, by which people with high satisfaction may be more likely to continue using interventions [26]. There is a strong imperative to accurately analyze satisfaction and app intervention use to determine the suitability of intervention content for meeting end users’ needs. Recommendations, including page rating functionality, the ability to record favorite pages within apps, and the use of technical solutions such as Google Tag Manager with iFrame technology, provide the opportunity to accurately assess users’ satisfaction with interventions and to link satisfaction with corresponding usage data. This is particularly important in the cancer field owing to the changing nature of information and resources available for people living with cancer and their caregivers. Adequate usage data will inform how to design and maintain e-interventions to meet the needs of people affected by cancer.

The ADUCI Framework was developed by applying the method to the ACE app study for adequate data cleaning. Data cleaning frameworks have been applied to other areas of health care technologies such as electronic medical records [27] to ensure accuracy in the data extracted. In this study, we compared manually extracted data automatically generated from usage tracking platforms and data obtained after the ADUCI Framework was applied. Manually extracted data showed a 16% increase in the number of logins compared to that identified by the ADUCI Framework. Applying the ADUCI Framework to duration of use provided strong evidence for the need to thoroughly clean and analyze manually extracted data. App usage when the ADUCI Framework was applied showed much less variance in duration of use. This was due to the removal of logins with no use and the standardization of lengthy uses with no navigation. For example, application of the framework resulted in a difference of approximately 55.5 hours of use of the *Cancer Information* section of the ACE app, with no navigation. Total duration of use of the app for the intervention period was 53,074 seconds when the ADUCI Framework was applied, which was recorded as approximately 72% higher (91,256 seconds) without the Framework. Combined with discrepancies evident in our report in which the duration of use in a specific app section exceeded the duration of login on several occasions, these findings highlight the inflation of results that can occur without the use of a framework. The use of the ADUCI Framework during development and testing highlight areas where data cleaning and analysis can be improved for future interventions.

Within the psycho-oncology setting, there is a need for cost-effective assessments of interventions [28] that meet users’ needs. Assessing usage data with the ADUCI Framework and applying the recommendations for practice outlined in this report may help to more accurately assess user engagement throughout the life cycle of an intervention and allow for a thorough analysis of cost-effectiveness [9]. This approach can continue after translating research into practice, which facilitates the ongoing upkeep of interventions and potential cost savings in being able to amend existing interventions rather than the cost of developing new interventions or undertaking audits.

### Limitations

This study was limited as the ADUCI Framework was developed from a sample of 43 participants who received the e-intervention. However, usage (over 250 logins) allowed for the Framework to be thoroughly tested in this cohort. A larger sample would enable verification of the Framework and a thorough comparison of observed use and expected use during user testing. With a larger sample, it may be possible to assess users’ behavior and provide additional information about the navigation patterns users follow.

### Conclusion

Accurate data usage analysis is vital in the growing eHealth environment to ensure that e-interventions are promoting engagement. In this study, we have proposed a framework to support the assessment of apps guided by user-centered design and human-computer interaction. This first iteration of the ADUCI Framework highlights how data can be accurately extracted, the potential for resource savings in a project life cycle, and provides recommendations for future studies to incorporate in their project design to enhance the usage data captured.

## Abbreviations

CCV: Cancer Council Victoria
eHealth: electronic health
ADUCI: Assessment of Data Usage of Cancer Electronic Interventions
